# Efficacy of a Novel Sigma-1 Receptor Antagonist for Oxaliplatin-Induced Neuropathy: A Randomized, Double-Blind, Placebo-Controlled Phase IIa Clinical Trial

**DOI:** 10.1007/s13311-017-0572-5

**Published:** 2017-09-18

**Authors:** Jordi Bruna, Sebastián Videla, Andreas A. Argyriou, Roser Velasco, Jesús Villoria, Cristina Santos, Cristina Nadal, Guido Cavaletti, Paola Alberti, Chiara Briani, Haralabos P. Kalofonos, Diego Cortinovis, Mariano Sust, Anna Vaqué, Thomas Klein, Carlos Plata-Salamán

**Affiliations:** 1 Hospital Universitari de Bellvitge-ICO L’Hospitalet, Barcelona, Spain; 2 Clinical Investigation, Laboratorios del Dr. Esteve, Barcelona, Spain; 3 University Hospital of Patras, Rion, Patras, Greece; 4 Medicxact, Alpedrete, Madrid, Spain; 5 Hospital Clinic, Barcelona, Spain; 6 University of Milano-Bicocca, Monza, Italy; 7 Azienda Ospedaliera di Padova, Padua, Italy; 8 San Gerardo Hospital, Monza, Italy; 9 Mundipharma Research GmbH & Co. KG, Limburg (Lahn), Germany

**Keywords:** Neurotoxicity, Neuropathic pain, Adverse effects, Chemotherapy, Colorectal cancer, MR309/E-52862

## Abstract

This trial assessed the efficacy of MR309 (a novel selective sigma-1 receptor ligand previously developed as E-52862) in ameliorating oxaliplatin-induced peripheral neuropathy (oxaipn). A discontinuous regimen of MR309 (400 mg/day, 5 days per cycle) was tested in patients with colorectal cancer receiving FOLFOX in a phase II, randomized, double-blind, placebo-controlled, multicenter clinical trial. Outcome measures included changes in 24-week quantitative measures of thermal sensitivity and total neuropathy score. In total, 124 patients were randomized (1:1) to MR309 or placebo. Sixty-three (50.8%) patients withdrew prematurely before completing 12 planned oxaliplatin cycles. Premature withdrawal because of cancer progression was less frequent in the MR309 group (7.4% *vs* 25.0% with placebo; *p* = 0.054). MR309 significantly reduced cold pain threshold temperature [mean treatment effect difference (SE) *vs* placebo: 5.29 (1.60)°C; *p* = 0.001] and suprathreshold cold stimulus-evoked pain intensity [mean treatment effect difference: 1.24 (0.57) points; *p* = 0.032]. Total neuropathy score, health-related quality-of-life measures, and nerve-conduction parameters changed similarly in both arms, whereas the proportion of patients with severe chronic neuropathy (National Cancer Institute Common Terminology Criteria for Adverse Events ≥ 3) was significantly lower in the MR309 group (3.0% *vs* 18.2% with placebo; *p* = 0.046). The total amount of oxaliplatin delivered was greater in the active arm (1618.9 mg *vs* 1453.8 mg with placebo; *p* = 0.049). Overall, 19.0% of patients experienced at least 1 treatment-related adverse event (25.8% and 11.9% with MR309 and placebo, respectively). Intermittent treatment with MR309 was associated with reduced acute oxaipn and higher oxaliplatin exposure, and showed a potential neuroprotective role for chronic cumulative oxaipn. Furthermore, MR309 showed an acceptable safety profile.

**Electronic supplementary material:**

The online version of this article (10.1007/s13311-017-0572-5) contains supplementary material, which is available to authorized users.

## Introduction

Chemotherapy-induced peripheral neuropathy (CIPN) is the most prevalent neurological complication of anticancer treatment and a common dose-limiting side effect [1]. Oxaliplatin (OXA) is the cornerstone of colorectal cancer treatment [2] and is being increasingly used to treat other malignancies. However, OXA-induced peripheral neuropathy (OXAIPN) is the most prominent toxicity both during and after the completion of chemotherapy, compromising therapeutic outcomes and patients’ functional capacity and quality of life [1, 3]. OXAIPN usually presents as 2 distinct clinical syndromes [4]. One is a classic cumulative, chronic sensory neuropathy, which involves typical features of platinum drug peripheral neuropathy. The other, more distinctive of OXA, is an acute transient syndrome characterized by paresthesias and dysesthesias triggered by exposure to cold in the distal extremities and the perioral region [5]. The acute syndrome can also include a neuromyotonia-like syndrome, with motor hyperexcitability symptoms [1].

The cumulative sensory neuropathy is driven by the capacity of platinum to form DNA adducts and crosslinks, oxidative stress, mitochondrial dysfunction, and increased p53, p38, and extracellular regulated kinase 1/2 activity in dorsal root ganglia neurons [1, 4]. In contrast, acute OXA-induced neuropathy is likely related to the dysfunction of the axonal nodal voltage-gated sodium channels, in which chelation of intracellular calcium by oxalate and the sensitization of transient receptor potential channels in dorsal root ganglia neurons play a role [4]. These effects ultimately lead to reduced axonal refractoriness and superexcitability [6–8].

Effective neuroprotective therapies against OXAIPN or CIPN have been largely sought. Unfortunately, none of the agents or therapeutic strategies tested to date has demonstrated unequivocal efficacy [9]. Only duloxetine has been shown to provide minor—albeit consistent and clinically relevant—relief of pain in patients with established cumulative OXAIPN [10].

MR309 (CAS registry number 1265917-14-3), previously developed as E-52862, is a novel selective sigma-1 receptor (S1R) antagonist. The S1R is a transmembrane protein found in the endoplasmic reticulum, specifically at the mitochondria-associated endoplasmic reticulum membrane, and has the ability to translocate to the plasma membrane [11]. In the nervous system, S1Rs mediate the regulation of several processes, such as neuritogenesis, the activity of potassium channels and *N*-methyl-d-aspartate receptors, and calcium homeostasis [11]. S1Rs have a modulatory role in nociception, and attenuate intracellular signal transduction cascades related to noxious stimuli and sensitization phenomena [12]. To date, MR309 is the only molecule of the new drug class of S1R antagonists that has progressed to clinical development [12]. In preclinical studies that included CIPN models, MR309 reduced hyperalgesic effects, as well as cold and mechanical allodynia [13, 14], and was able to prevent the early ultrastructural mitochondrial changes observed in CIPN [15].

The OXAIPN is a valid disease model to test the modulatory effect of S1R antagonists over axonal membrane excitability and their neuroprotective potential. Based on the good safety, tolerability, and pharmacokinetic properties of MR309 at doses up to 400 mg for 8 consecutive days in phase I clinical studies [16], a proof-of concept phase II clinical trial was designed with the aim of testing the suitability of MR309 to prevent OXAIPN. To our knowledge, this is the first report on the neuroprotective efficacy of a S1R antagonist in a clinical trial on CIPN.

## Methods

### Study Design

This was a proof-of-concept, phase II, randomized (1:1), double-blind, placebo-controlled, parallel-group clinical trial. It was carried out in 5 major hospitals in Europe (2 in Spain, 2 in Italy, and 1 in Greece). The ethics committees/institutional review boards of the participating hospitals approved the study protocol prior to starting recruitment.

The patients were assessed using both patient-reported outcomes and objective physician-assessed endpoints.

A synopsis of the protocol and the major results of the trial are available at: https://www.clinicaltrialsregister.eu/ctr-search/search?query=eudract_number:2012-000398-21.

### Patients

Chemotherapy-naïve patients (aged 18–80 years) with colorectal cancer diagnosed within the last 2 years and scheduled to receive OXA within a FOLFOX chemotherapeutic regimen were eligible. The main inclusion criteria were having a planned OXA dose ≥ 60 mg/m^2^ in the first cycle, a Karnofsky performance status score ≥ 70, and a peripheral sensory neuropathy toxicity grade assessed with the National Cancer Institute Common Terminology Criteria for Adverse Events (NCI-CTCAE) of ≤ 1 at recruitment. Patients with neurological conditions that might interfere with the evaluation of the study objectives, receiving medications with potential pharmacologic interactions with the study drug (listed in the protocol), or with a life expectancy < 4 months were excluded. All patients provided written informed consent to participate.

### Randomization and Masking

Patients and investigators were blinded to the allocation. Patients were randomly assigned to either MR309 or placebo following a computer-generated sequence of random permutations of 2 elements in blocks of 4. Randomization was stratified according to the type of chemotherapeutic regimen (FOLFOX 4 or 6 modified, consisting of OXA 85 mg/m^2^ infusion on day 1 combined with leucovorin and 5-fluorouracil, in a 2-week cycle). Individually sealed opaque envelopes were provided in case the investigators needed to know the allocation of a particular patient in a medical emergency. MR309 and placebo tablets had identical appearance.

### Procedures

**Fig. 1:**
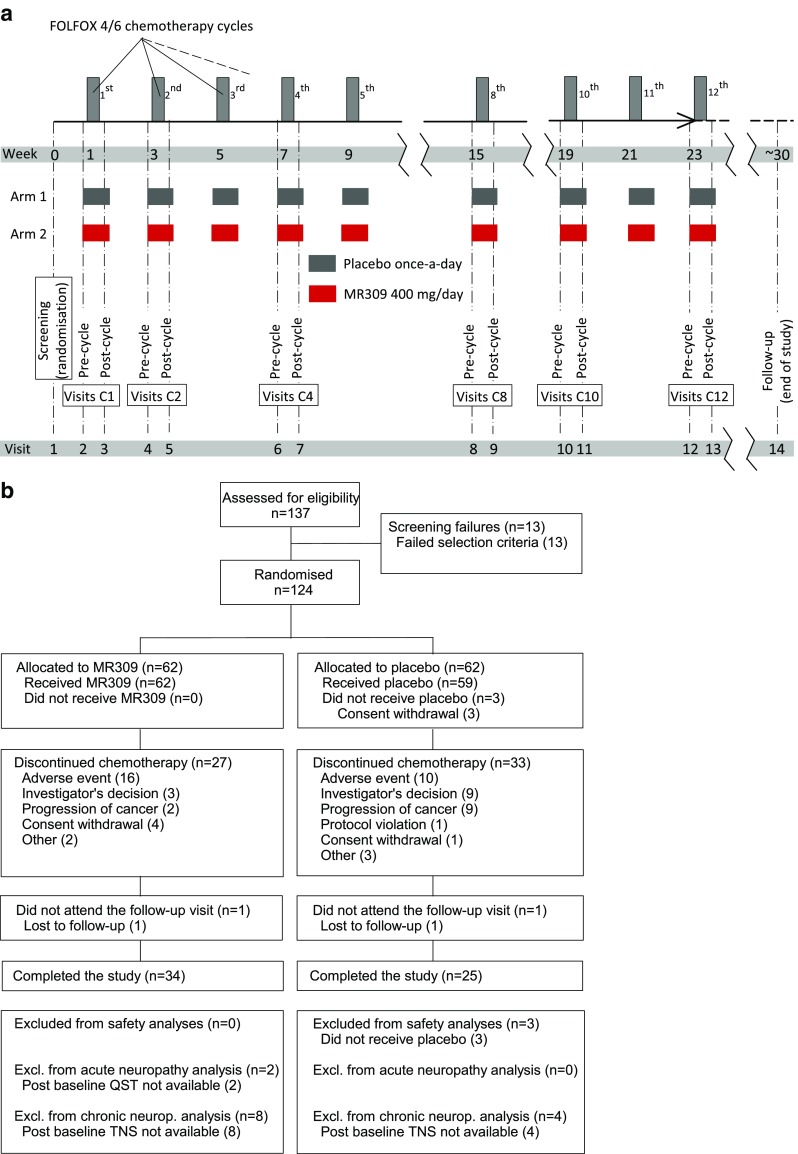
**a** Diagram of the study design; **b** trial profile (CONSORT diagram). TNS = total neuropathy score; QST = quantitative sensory testing

Patients received 1 oral daily dose of the study drug or placebo during the first 5 days of each chemotherapy cycle, up to a maximum of 12 cycles (Fig. 1a). In the active group, each tablet contained 400 mg MR309. This intermittent, rather than continuous dosing regimen was decided because repeated dosing schedules > 8 days had not been tested by the time of protocol development. During chemotherapy, 2 visits were done during chemotherapy cycles 1, 2, 4, 8, 10, and 12. The first visit occurred 1 day before the planned start of chemotherapy infusions (precycle assessment) and the second between 24 and 48 h following the end of OXA infusion. A follow-up visit was done 6 weeks after the end of chemotherapy.

### Outcomes

In this proof-of-concept study, no primary and secondary endpoints were predefined. The relative degree of acute OXAIPN syndrome was quantitatively assessed by changes of cold pain perception *versus* baseline, providing the cold pain threshold (CPT) and the intensity of pain evoked by suprathreshold cold stimuli on the skin covering the thenar eminence. Both were determined with the method of limits from a resting temperature of 32°C with the Thermal Sensory Analyser II by Medoc [for a more complete description of this quantitative sensory testing (QST) procedure, see [17]]. Evoked pain was measured in both the dominant and nondominant hands. In addition, the warm detection threshold (WDT) and cold detection thresholds (CDT) were measured by QST to monitor acute neuropathy of non-nociceptive small nerve fibers. Thermal QST is specially suitable for the diagnosis of small-fiber neuropathy that cannot be assessed by standard nerve conduction studies (NCS) [18]. The incidence of sensory and motor signs and symptoms of acute OXAIPN were measured by the OXA Neuropathy Questionnaire (OXA-NQ) as used previously [5]. Cumulative OXAIPN was assessed with the clinical version of the total neuropathy score (TNS; score range 0–28, with higher values indicating more severe neuropathy) [19]; the grade of peripheral sensory neuropathy toxicity was assessed with the NCI-CTCAE scale, NCS and patient-reported outcome measures from the European Organization for Research and Treatment of Cancer (EORTC). The doses of OXA delivered throughout the study were also recorded. Safety was analyzed as the incidence of adverse events (AEs) by severity, AEs leading to withdrawal, and AEs related to the study drug.

### Statistical Analyses

This was a proof-of-concept exploratory study. Therefore no adjustment by multiplicity was made to account for the various endpoints considered. Sample size was determined to detect, with a power of 80%, a difference of 7.1 points between study group means for TNS at the end of chemotherapy [20]. Sample size was calculated based on this endpoint, as it required larger sample sizes than QST-based endpoints (internal calculations based on the results of a previous study by the authors [17]). The evolution of QST-based endpoints and of the TNS was compared between study arms using generalized linear mixed models for longitudinal data with the study group and site as fixed factors, the baseline value as covariate, and the patient as a random factor. Some models were also adjusted by the total accumulated amount (mg) of OXA delivered. Transversal comparisons (at specific time points) between study groups were done using Mann–Whitney tests for continuous endpoints and either Pearson’s χ^2^ or Fisher’s exact tests for proportions. Time-to-event endpoints (time to withdrawal, the duration of signs and symptoms of acute neuropathy, and time to first occurrence of grade ≥ 3 NCI-CTCAE neuropathy) were described using the Kaplan–Meier method and compared by means of log-rank tests. Efficacy analyses were done on an intention-to-treat basis, using the data from all patients who had a baseline evaluation and at least 1 postbaseline assessment available. For sensitivity analyses, descriptions and inferences were repeated on a per-protocol set. Safety was analyzed on patients who received at least 1 dose of the study drug. Some post-hoc analyses were performed to further explore the beneficial effects that were observed in the acute neuropathy. These are marked as such in the exposition of results. All analyses were done with the version 9.1.3 of the statistical package SAS (SAS Institute, Cary, NC, USA).

## Results

### Patient Characteristics and Disposition

Between 27 September 2012 and 4 April 2014, 137 patients were recruited, of whom 124 (61 from Spain, 14 from Italy, and 49 from Greece) were randomized (62 to MR309, 62 to placebo) (Fig. 1b). Baseline demographic characteristics were similar between the groups (Table 1). Although the average number of cycles and the incidence of OXA dose reductions were similar in both groups, the total accumulated amount of OXA delivered was greater in the MR309 group. The difference only reached statistical significance for the raw dose (1618.9 mg *vs* 1453.8 mg with placebo; *p* = 0.049), not for the body surface area-adjusted dose (911.0 mg/m^2^ *vs* 822.3 mg/m^2^ with placebo; *p* = 0.062). Despite being lower with MR309, the proportion of patients who withdrew prematurely did not differ significantly between the groups [27/62 (43.5%) *vs* 36/62 (58.1%); *p* = 0.106]. However, this difference was nearly significant for premature withdrawals due to cancer progression [2/27 (7.4%) *vs* 9/36 (25.0%); *p* = 0.054]. Approximately half of patients (50.4%) had advanced cancer (stages ≥ IIIC): 53.2% in the MR309 group and 47.5% in the placebo group. Forty patients (32.3%) had metastatic disease.

**Table 1:** Patients’ baseline characteristics in the safety analysis set

	MR309 (*n* = 62)	Placebo (*n* = 59)
Sex		
Female	25 (40.3)	21 (35.6)
Male	37 (59.7)	38 (64.4)
Median (range) age (y)	61 (24–75)	62 (27–79)
Mean ± SD BMI (kg/m^2^)	25.3 ± 4.5	25.4 ± 3.5
Stage of colorectal cancer		
I	0	1 (1.7)
IIA	4 (6.5)	3 (5.1)
IIB	4 (6.5)	3 (5.1)
IIC	5 (8.1)	4 (6.8)
IIIA	2 (3.2)	2 (3.4)
IIIB	14 (22.6)	18 (30.5)
IIIC	12 (19.4)	9 (15.3)
IVA	8 (12.9)	8 (13.6)
IVB	13 (21.0)	11 (18.6)
Metastatic disease		
Yes	21 (33.9)	19 (32.2)
Chemotherapy regimen		
FOLFOX 4	24 (38.7)	23 (39.0)
FOLFOX 6	38 (61.3)	36 (61.0)
Mean ± SD number of chemotherapy cycles received	9.7 ± 3.7	9.5 ± 3.1
Incidence of OXA dose reductions	27 (43.5)	20 (33.9)
Mean ± SD total accumulated amount of OXA delivered (mg)	1618.9 ± 303.5^a^	1453.8 ± 405.1^a^

Data are *n* (%) unless otherwise indicated

BMI = body mass index; OXA = oxaliplatin

^a^Values for the full analysis set

### Effects on Acute OXAIPN

**Fig. 2:**
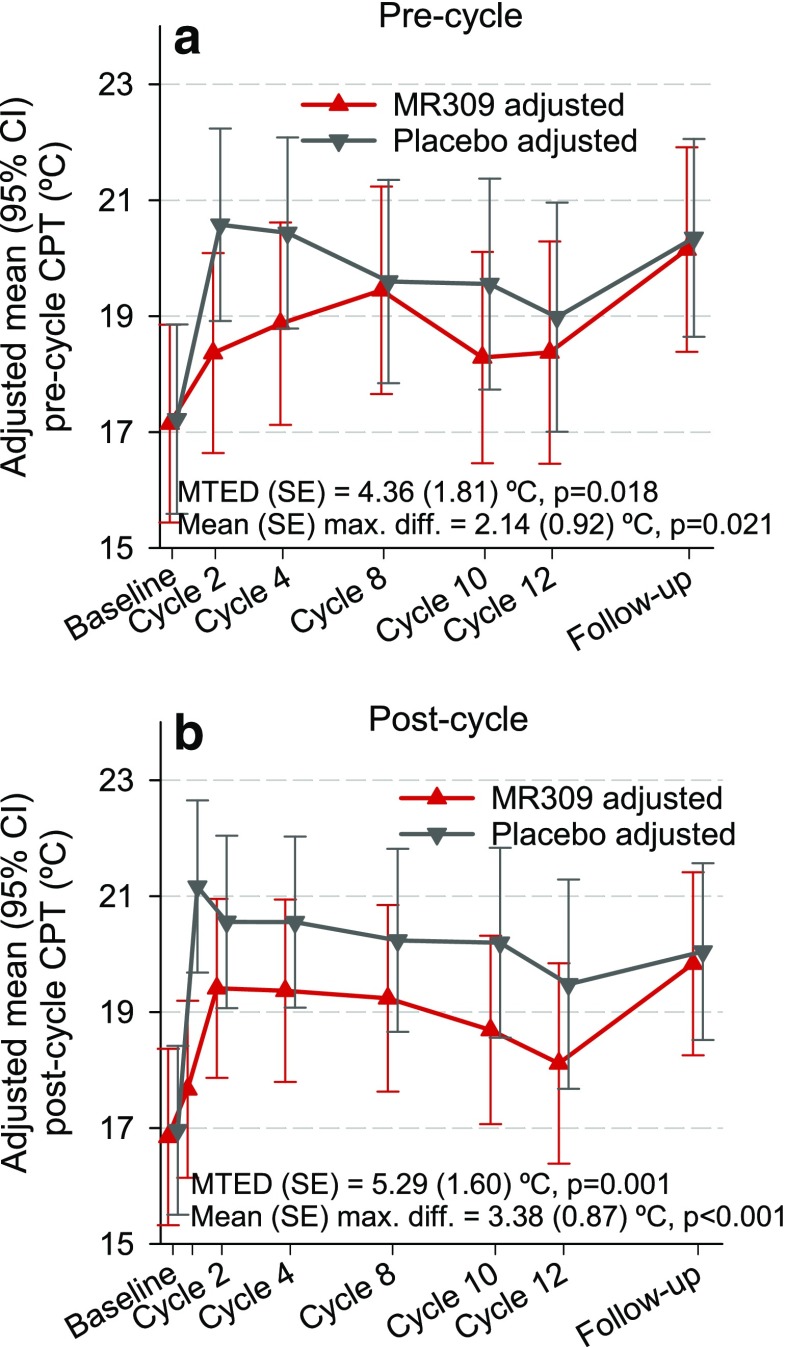
Evolution of cold pain threshold (CPT) determined either 1 day before the start of chemotherapy infusions (precycle) or 24–48 h following the end of oxaliplatin (OXA) infusion (postcycle). Data are least square means and 95% confidence intervals (CI) from the generalized linear mixed models. The sign of the difference is such that, if positive, it indicates a more favorable biological status with MR309 with respect to placebo. CPT = cold pain threshold, MTED = mean treatment effect difference (overall longitudinal measure of the difference between treatments throughout the study)

**Fig. 3:**
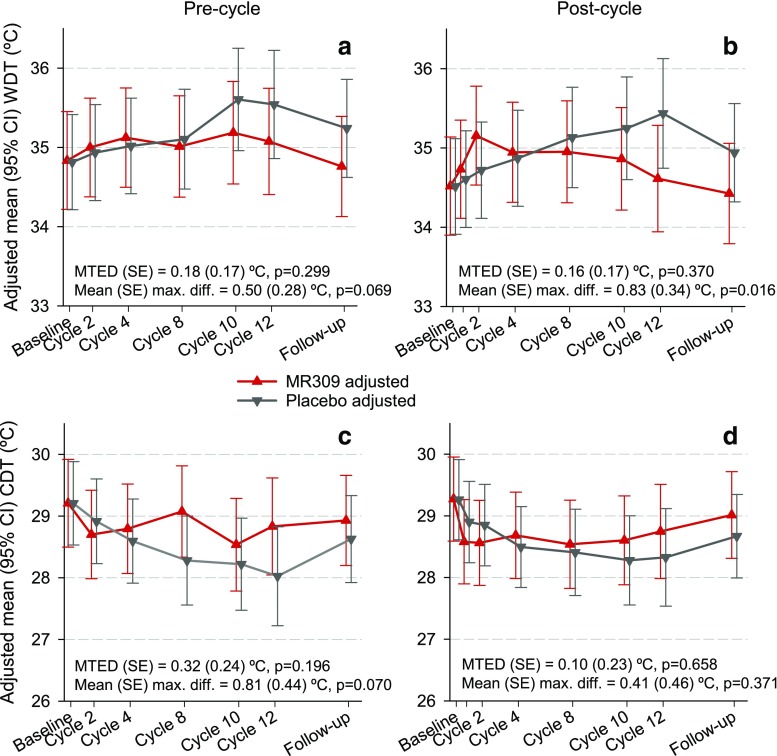
Evolution of thermal detection thresholds determined either 1 day before the start of chemotherapy infusions (precycle) or 24–48 h following the end of oxaliplatin (OXA) infusion (postcycle). Data are least square means and 95% confidence intervals (CI) from the generalized linear mixed models. The sign of the difference is such that, if positive, it indicates a more favorable biological status with MR309 with respect to placebo. WDT = warm detection threshold; CDT = cold detection threshold; MTED = mean treatment effect difference (overall longitudinal measure of the difference between treatments throughout the study)

**Fig. 4:**
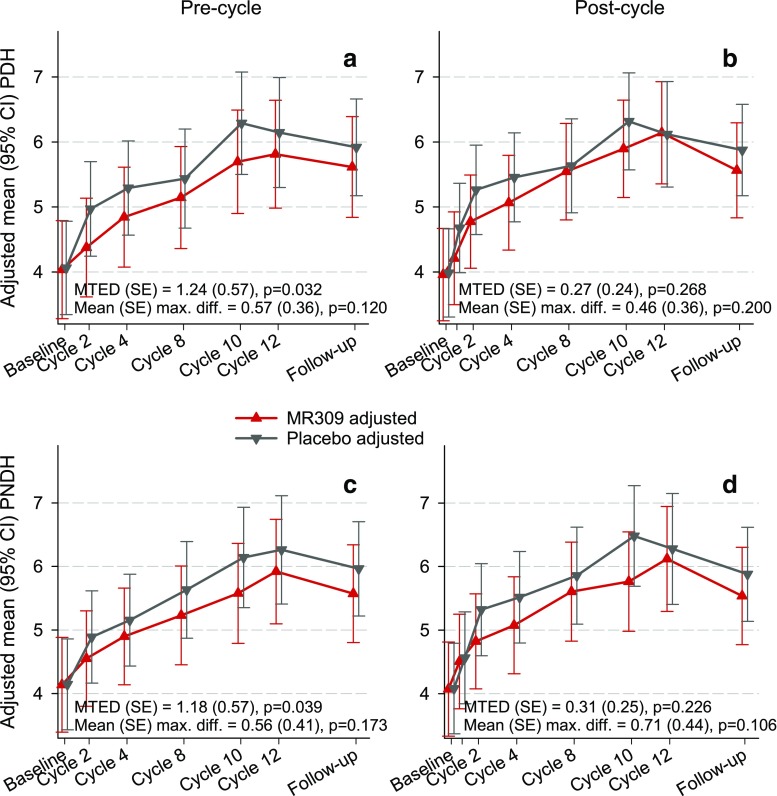
Evolution of the intensity of pain evoked by suprathreshold cold stimuli either 1 day before the planned start of chemotherapy infusions (precycle) or 24–48 h following the end of oxaliplatin (OXA) infusion (postcycle). Data are least square means and 95% confidence intervals (CI) from the generalized linear mixed models. The sign of the difference is such that, if positive, it indicates a more favorable biological status with MR309 with respect to placebo. PDH = intensity of pain evoked by suprathreshold cold stimuli in the dominant hand; PNDH = intensity of pain evoked by suprathreshold cold stimuli in the nondominant hand; MTED = mean treatment effect difference (overall measure of the difference between treatments throughout the study)

Figures 2, 3, and 4 show the evolution of CPT, thermal detection thresholds, and suprathreshold cold-evoked pain measures throughout the study. There were significant differences between the study groups favoring MR309 in the evolution of CPT at both the precycle and postcycle assessments, and the intensity of the pain evoked by suprathreshold cold stimuli at precycle assessments. As expected, after the first chemotherapy cycle, the temperature at CPT abruptly raised (cold was perceived as painful at higher temperatures) in the placebo group, indicating the induction of cold pain hyperalgesia after the first OXA application, whereas in the MR309 group the increase was more subtle. Just after the first cycle, the mean raw difference was above 3°C, favoring MR309 (Fig. 2b). The mean treatment effect differences (MTEDs) were even greater, around 5°C (Fig. 2a, b). A discrete thermal hypoesthesia developed throughout chemotherapy in the placebo group, as denoted by the progressive separation of WDT and CDT from the resting temperature (Fig. 3). Although the MTEDs for WDT and CDT were not significant, the between-group difference of WDT significantly favored the MR309 group at cycle 12 (Fig. 3b).

The intensity of cold-evoked pain increased as chemotherapy progressed and declined during the follow-up period in both study groups, but the increase was more pronounced in the placebo group up to the second cycle (Fig. 4). The resulting separation, which reached 0.5 points in a 10-point pain scale at some visits, was significant in the precycle measurements (Fig. 4a, c). In fact, the MTEDs were > 1 point over in the 10-point pain scale.

The pre- to postcycle changes were unfavorable in both groups. However, interpretation is challenging because this assessment is strongly influenced by what occurred from the previous cycle (e.g., in some instances the level of pain rose less in the placebo group than in the MR309 group because patients on placebo started from a much higher pain level at the precycle visit resulting from a more pronounced worsening from the previous cycle). For brevity, these results are not included, but a complete set of illustrative figures is available on request.

**Fig. 5:**
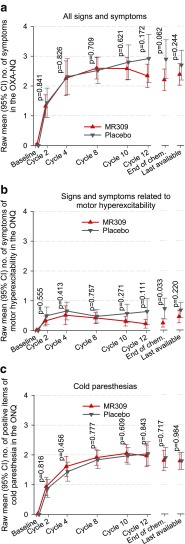
Evolution of the number of symptoms of acute neuropathy as reported by patients in the oxaliplatin (OXA)-Neuropathy Questionnaire (OXA-NQ). The values at the end of chemotherapy were taken during the last chemotherapy cycle regardless of whether it was cycle 12 or before (for patients who withdrew prematurely). The *p*-values were calculated for the null hypothesis that the number of symptoms was the same in both study arms using Mann–Whitney tests at each of the study visits. CI = confidence interval; ONQ = OXA-NQ

The mean count of signs and symptoms of acute neuropathy as reported by patients in the OXA-NQ was similar in both study groups up to cycle 10 (Fig. 5). At cycle 12 and at the end of chemotherapy—regardless of whether it was cycle 12 or before—the count was somewhat lower in the MR309 group, yet the differences did not reach statistical significance (Fig. 5a). However, the post-hoc analysis of 7 signs and symptoms related to motor hyperexcitability yielded average counts that were consistently lower in the MR309 group, and this difference reached statistical significance at the end of chemotherapy (Fig. 5b). The mean count of sensory symptoms (3 items regarding cold-induced paresthesia) was similar in both groups (Fig. 5c). Overall, all signs and symptoms included in the OXA-NQ lasted longer in the placebo group than in the MR309 group, yet the differences reached statistical significance only for difficulties in swallowing at cycle 12 (*p* = 0.023). A complete set of Kaplan–Meier plots is available on request.

### Effects on Cumulative OXAIPN

**Fig. 6:**
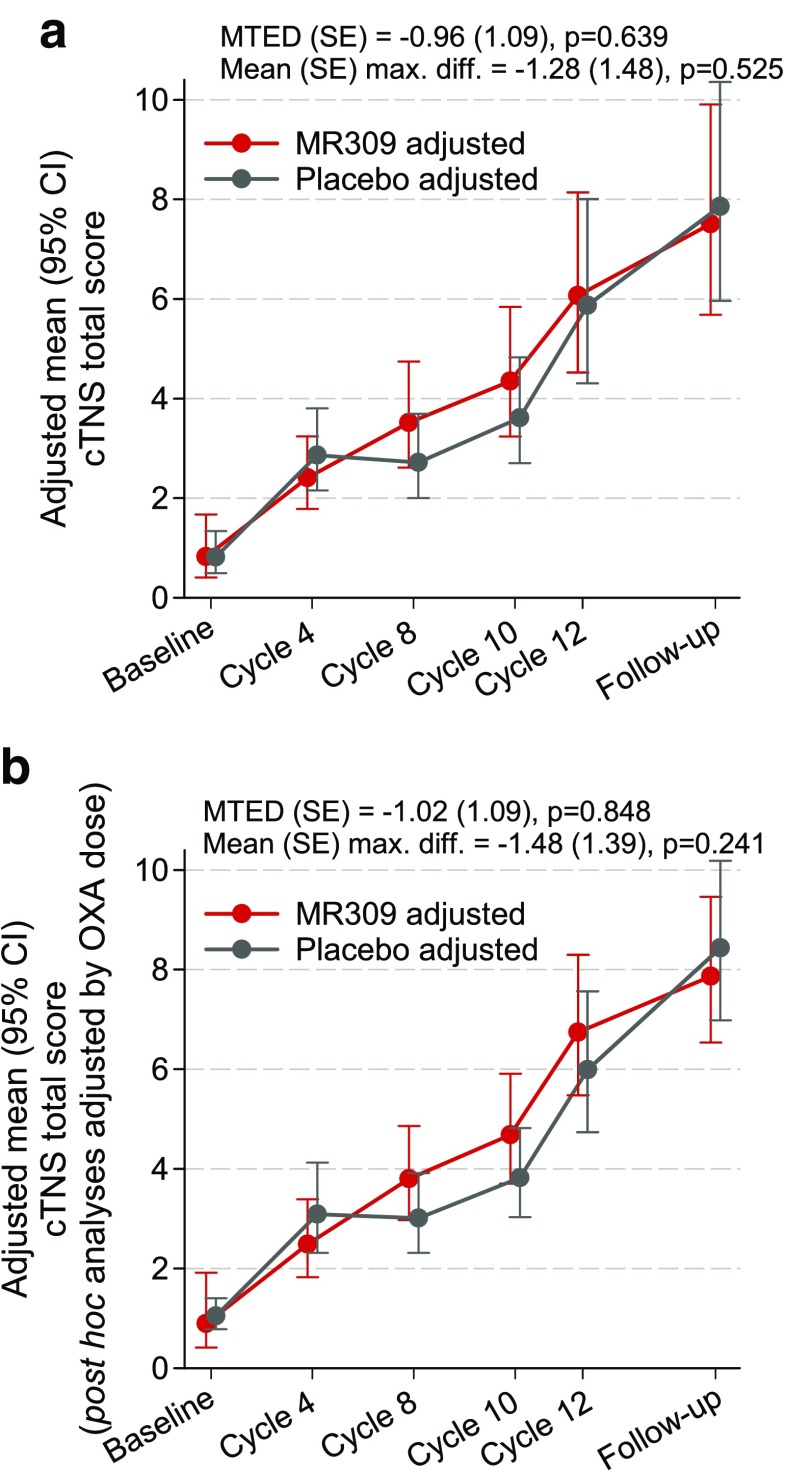
Evolution of clinical total neuropathy score (cTNS). Data are least square means and 95% confidence intervals (CI) from the generalized linear mixed models. The sign of the difference is such that, if positive, it indicates a more favorable biological status with MR309 with respect to placebo. MTED = mean treatment effect difference (overall measure of the difference between treatments throughout the study); OXA = oxaliplatin

The TNS showed progressive impairment throughout the study that was comparable in both groups (Fig. 6). The MTEDs were trivial both in a model that adjusted by baseline differences (Fig. 6a) and in a post-hoc model that additionally adjusted by the total amount of OXA delivered (Fig. 6b). Treatment effects were not significant.

**Fig. 7:**
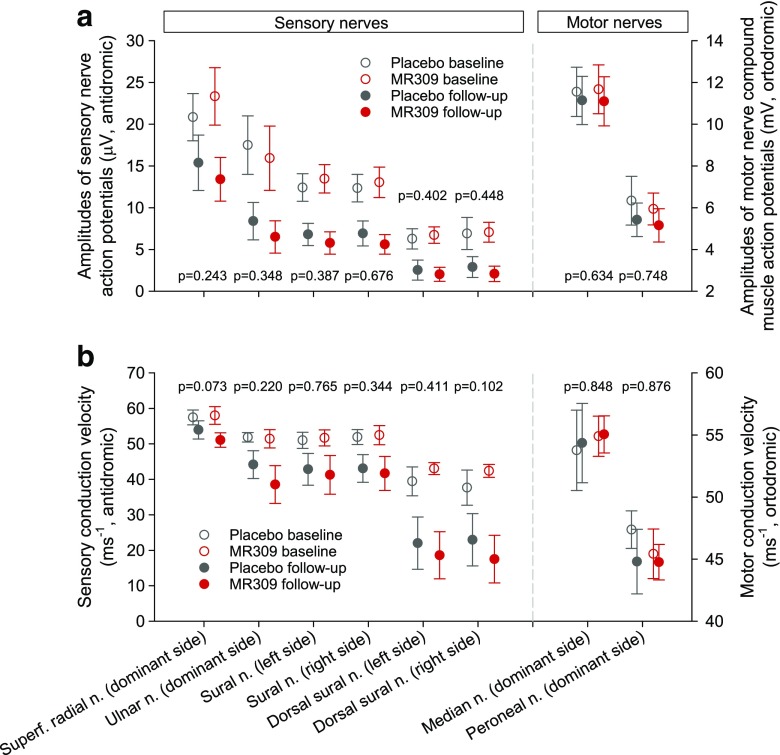
Amplitudes of action potentials and conduction velocities of the sensory and motor nerves assessed at the baseline and follow-up visits. Data are means and 95% confidence intervals. The *p*-values are from comparisons between study arms of either the values at the follow-up visit adjusted by baseline values (analysis of covariance for Gaussian data) or the changes from baseline (Mann–Whitney for non-Gaussian data).

On average, the grade of peripheral sensory neuropathy as per the NCI-CTCAE increased in parallel in both groups throughout the study. The proportions of patients who developed grade 2 or worse toxicity up to the follow-up assessment did not differ significantly between the groups [37/46 MR309 patients evaluated, 80.4% *vs* 28/41 placebo patients evaluated, 68.3% (*p* = 0.193)]. Nonetheless, the proportion of patients who showed grade 3 (severe) or worse toxicity was significantly lower in the MR309 group compared with the placebo group [1/33 patients evaluated, 3.0% *vs* 6/33 patients evaluated, 18.2% (*p* = 0.046)]. In contrast, other efficacy endpoints for cumulative OXAIPN varied in line with the TNS. The measures of sensory nerve conduction (Fig. 7) and the EORTC measures of health-related quality of life (data available on request) declined similarly in both groups throughout the study. Treatment effects were nonsignificant in all instances.

### Safety

The proportion of patients with at least 1 AE related to study drug was higher in the MR309 group (25.8% in the MR309 group *vs* 11.9% in the placebo group; *p* = 0.051) (Table 2). Nausea, diarrhea, mucositis, decreased appetite, dysgeusia, and oral paresthesia were slightly more frequent in the MR309 group. Among treatment-related AEs, only some concerning the nervous system [dizziness, headache and neurotoxicity (2 patients each)] were more common in the MR309 group (Table 2).

**Table 2:** Summary of adverse events (AEs) in the safety analysis set

	MR309 (*n* = 62)	Placebo (*n* = 59)	*p*-value
Patients with any AE^a^	62 (100.0)	58 (98.3)	0.488^a^
Patients with any SAE	9 (14.5)	9 (15.3)	0.909^b^
Patients with any SAE	21 (33.9)	26 (44.1)	0.250^b^
Patients with any AE related to study drug^b^	16 (25.8)	7 (11.9)	0.051^b^
Patients with any severe and related AE	4 (6.5)	3 (5.1)	0.519^a^
Patients with AEs leading to withdrawal	16 (25.8)	10 (16.9)	0.236^b^
Most common AEs^c^			
Asthenia	30 (48.4)	28 (47.5)	0.919^b^
Paraesthesia	30 (48.4)	27 (45.8)	0.773^b^
Neutropenia	31 (50.0)	24 (40.7)	0.303^b^
Thrombocytopenia	25 (40.3)	28 (47.5)	0.429^b^
Nausea	27 (43.5)	22 (37.3)	0.483^b^
Diarrhea	25 (40.3)	21 (35.6)	0.592^b^
Mucosal inflammation/mucositis	21 (33.9)	14 (23.7)	0.219^b^
Decreased appetite	18 (29.0)	14 (23.7)	0.509^b^
Dysgeusia	19 (30.6)	13 (22.0)	0.283^b^
Oral paraesthesia	17 (27.4)	14 (23.7)	0.642^b^
Most common related AEs^d^			
Nausea	5 (8.1)	2 (3.4)	0.440^a^
Neutropenia	1 (1.6)	3 (5.1)	0.356^a^
Thromobocytopenia	1 (1.6)	2 (3.4)	0.613^a^
Diarrhea	2 (3.2)	1 (1.7)	1.000^a^
Asthenia	1 (1.6)	2 (3.4)	0.613^a^
Hypokalaemia	0	3 (5.1)	0.113^a^
Vomiting	2 (3.2)	0	0.496^a^
Fatigue	1 (1.6)	1 (1.7)	1.000^a^
Dizziness	2 (3.2)	0	0.496^a^
Headache	2 (3.2)	0	0.496^a^
Hypoesthesia	1 (1.6)	1 (1.7)	1.000^a^
Unspecified neurotoxicity	2 (3.2)	0	0.496^a^

Data are *n* (%) unless otherwise indicated. Prior to analysis, adverse events were coded with the Medical Dictionary for Regulatory Activities

SAE = serious adverse event

^a^Fisher’s exact test

^b^Pearson’s χ^2^ test

^c^Present in at least 25% of patients

^d^Present in at least 1% of patients

The sensitivity analyses (in the per-protocol set) yielded similar results to the main analyses, with the exception that the treatment effect over the intensity of cold-evoked pain in the nondominant hand in favor of MR309 was nonsignificant (data available on request).

## Discussion

The selective S1R antagonist MR309 was able to partially preserve the CPT, reduce cold-evoked pain, and motor hyperexcitability signs and symptoms in patients with colorectal cancer treated with OXA. In contrast, the efficacy against cumulative OXAIPN was inconsistent as clinical benefits were just observed with one of the tools employed. Nevertheless, patients in the active group were able to receive a higher accumulated amount of OXA. In addition, MR309 showed acceptable safety and tolerability profiles. These results suggest that this first-in-class drug with a novel mechanism of action improves some symptoms and signs of acute OXAIPN.

At baseline, CDT and WDT, between 3–4°C and 2–3°C below and above the resting temperature of 32°C, respectively, were normal and consistent with those reported in other studies [21–23]. The CPT was closer to resting temperatures compared with what was reported in these studies but still within the wide range of response of C polymodal nociceptors [24] and within the range reported in a review of previous studies [22]. During the initial chemotherapy cycles, when the confusion brought by structural axonal damage or loss secondary to platinum deposition in neuronal bodies is not expected, patients treated with MR309 showed significantly less cold allodynia (CPT closer to baseline levels) and cold hyperalgesia (lower intensity of cold-evoked pain) than patients treated with placebo. Non-nociceptive cold perception primarily relies on specialized Aδ fibers that form cold-sensitive free terminals in the skin and project into the cold pathways sensing innocuous cold stimuli (response range between 17°C and 40°C), and on Aδ fiber type I mechano-heat nociceptors and C polymodal nociceptors that sense noxious cold (response range between −10°C and 20°C) [24]. Since a dynamic opposition has been described between activity in the cold sensitive and nociceptive pathways [25], the simultaneous reduction of the excitability in both paths by MR309 would be required to produce the observed effects.

Despite the fact that acute OXAIPN rarely led to treatment withdrawal in this study, several findings stress the importance of discovering a novel, effective agent to prevent it. Firstly, there is evidence suggesting that acute OXAIPN is linked to the occurrence and severity of the cumulative neuropathy [6, 7, 26, 27]. Secondly, although the clinical relevance of the reduction of cold-evoked pain for the tested patients is unknown, MR309 was able, even in a nonintensive dosing regimen, to improve both objective physical parameters and subjective clinical indicators of acute OXAIPN.

However, the results do not consistently support the neuroprotective potential of this agent on cumulative OXAIPN. Although the TNS has been regarded as useful for measuring the severity of chronic peripheral toxic neuropathies caused by cytostatic agents [28] and showed higher sensitivity to CIPN effects than the NCI-CTCAE scale [19], only the latter instrument showed the superiority of MR309 over placebo in reducing the transition from grade 2 to grade 3 or higher chronic neuropathy. The values of the TNS in this study were similar to those reported in a large series of patients who received comparable OXA schedules [27]. Likewise, the proportion of patients who developed severe neuropathy according to NCI-CTCAE in our placebo group was comparable to the incidence reported previously in patients treated with OXA [6, 27] but was significantly lower in the MR309 group. Why the TNS was not sensitive to the MR309 effects sensed with the NCI-CTCAE toxicity scale is not clear. The TNS is a global measure of peripheral nerve function that entails a range of neurological examinations, but it is not specifically devoted to sensory symptoms. Furthermore, it places much emphasis on the spatial distribution and extension of sensory alterations (4/7 items), so that severity is virtually equated to extension, whereas the NCI-CTCAE is chiefly a subjective assessment of the functional impact of the symptoms of neuropathy [19].

Whether or not functional limitations were key in the disparity was not clarified by the results obtained for the health-related quality-of-life measures, as these did not differ between the study groups. Although the measure used in this study (the EORTC-CIPN20) is one patient-reported outcome that has gained wide recognition for CIPN assessment, it is still under development, and a very recent report has raised concerns about its psychometric properties [29]. Thus, technical issues might have contributed to inconclusive results. Also, a longer time lapse might be required to sense QoL compromises caused by peripheral neuropathy [3, 30]. Neither of the NCS provided additional information, as they found comparable declines in the amplitudes of action potentials and the conduction velocities in both study groups. These findings are consistent with the scarce correlation reported between objective physician-assessed measures and the CIPN symptoms reported by patients [31].

In addition to the large-fiber sensory neuropathy usually associated with OXAIPN, the relative warm hypoesthesia that developed during anticancer treatment in the placebo group may be interpreted as a discrete cumulative small-fiber neuropathy, which is in line with histological findings on intraepidermal nerve-fiber density [32].

The accumulated dose of OXA was significantly greater in the MR309 group. This may have also contributed to blunting of the differences between groups at the end of the study, because the severity of OXAIPN is related to the dose delivered [1]. Speculatively, MR309 might have improved the tolerability of OXA, allowing a greater exposure before the signs and symptoms of neuropathy leveled in accordance with those observed in the placebo group. In turn, augmenting the exposure to OXA might improve the antineoplastic efficacy of the chemotherapy. It is also worth noting that some concerns have been raised regarding the potential effects of neuropathy prevention drugs on the antitumor properties of OXA, in particular the liability to reduce its therapeutic activity [33]. This study found no evidence of such reduction. On the contrary, fewer patients in the MR309 group withdrew from the study prematurely because of cancer progression than in the placebo group.

MR309 was well tolerated. As expected in patients with cancer receiving chemotherapy, nearly all had AEs during the study, but only a small fraction was related to the investigational drug. With the exception of neutropenia and thrombocytopenia (one patient each; Table 2), no related AE was unexpected, unanticipated, or had unusual severity. Special attention should be paid to hematologic AEs in future studies of MR309.

The major limitation of this proof-of-concept study lies in its exploratory nature. Since no primary and secondary endpoints were predefined, it should be regarded as hypothesis-generating, inductive research. Because several measures of OXAIPN were compared between groups, multiplicity issues might have compromised the type I error rate. Therefore, the observed protection MR309 offered against acute neuropathy, including symptoms of sensory and motor hyperexcitability, as well as severe cumulative neuropathy must be confirmed in larger, dedicated studies.

We conclude that the selective S1R antagonist, MR309, reduced acute OXAIPN (cold pain and motor symptoms), allowed patients to be exposed to higher doses of OXA, and was well tolerated. The effects on cumulative neuropathy were unclear; however, the reduced incidence of grade 3 toxicity and the known link between the acute and chronic syndromes provides a basis for further exploration of the full potential of MR309 in the CIPN setting. This should entail the assessment of different regimens of administration and continuous dosing during the full chemotherapy period. To our knowledge, this is the first report of efficacy of an agent specifically targeted towards putative pathophysiological mechanisms involved in neuronal hyperexcitability and neurotoxicity. Given the lack of innovation in the development of neuropreventive drugs in recent decades [9], the discovery of a new agent based on novel molecular targets may represent a relevant medical progress.

## Electronic supplementary material

ESM 1 (PDF 498 kb)
